# Astrocytoma development following complete multiple myeloma remission in a 49-year-old patient: A case report

**DOI:** 10.3892/etm.2013.1179

**Published:** 2013-06-25

**Authors:** XIAOYAN HAN, DIAN JIN, GAOFENG ZHENG, YI LUO, ZHEN CAI

**Affiliations:** Multiple Myeloma Center, Bone Marrow Transplantation Center, Department of Hematology, The First Affiliated Hospital of Medical College, Zhejiang University, Hangzhou, Zhejiang 310003, P.R. China

**Keywords:** multiple myeloma, astrocytoma development, intracranial space-occupying lesion, cancer, bone marrow

## Abstract

Multiple myeloma (MM), one of the B-cell non-Hodgkin lymphomas, is a bone marrow-derived, antibody-producing cancer of the plasma cells. In the advanced stages, the cancer cells frequently cause widespread osteolytic bone damage; however, in rare cases, MM also manifests as an intracranial plasmacytoma. In the present study, we describe a case in which a patient, initially treated for MM and with subsequent complete remission, was admitted to hospital with a lesion in the right cerebellar hemisphere and neurological symptoms of a brain tumor. Our initial diagnosis was an MM relapse with the rare occurring intracranial manifestation. However, pathological tests confirmed the diagnosis of a high-grade astrocytoma. In this case report, we describe the characteristics, as well as the treatment issues, diagnoses and clinical developments of this patient.

## Introduction

Multiple myeloma (MM) accounts for ∼10% of hematological malignancies (1). Despite the development of novel drug treatments and the advances in stem cell transplantation, which have improved survival rates, MM remains a difficult disease to cure. The involvement of the central nervous system (CNS) in MM is rare, occurring in ∼1% of patients (2). In these instances, MM manifests as primary brain lesions, in the absence of initial systemic MM and without appearing as a complication of systemic MM. Gliomas account for 30–40% of all brain tumors (3). The World Health Organization (WHO) divides astrocytomas into four grades, of which the WHO Grades III (anaplastic astrocytoma) and IV (glioblastoma multiforme) are the malignant subtypes (3). These are invasive primary brain tumors that are difficult to treat and that exhibit a rapid proliferation rate. Resection and radio- or chemotherapies are the commonly accepted standard treatments, although novel agents are being tested in clinical trials. The coexistence of malignant astrocytoma and MM is exceedingly rare, with few cases documented and, therefore, when a patient with MM develops intracranial space-occupying lesions, the first diagnostic assumption is an intracranial MM tumor, rather than a primary brain tumor, such as astrocytoma. However, since the treatment and prognosis of intracranial plasmacytomas and astrocytomas differ, a definite differential diagnosis is imperative. In the current study, we describe a case of a 49-year-old patient with MM, who subsequently developed an anaplastic astrocytoma. In addition, we discuss the importance of a differential diagnosis between intracranial plasmacytoma and astrocytoma, as well the correlation between MM and astrocytoma.

## Case report

### Primary treatment of the patient

A 49-year-old male was admitted to our hospital (The First Affiliated Hospital of Zhejiang University School of Medicine, Hangzhou, China) due to bone pain. The medical history of the patient revealed no notable events or symptoms of fatigue, weakness or recurrent infection during the preceding months, and there was no evidence of mental or neurological impairment. The results of a physical examination were as follows: total protein level, 73.2 g/l (normal range, 60.0–83.0 g/dl); albumin level 51.1 g/l (normal range, 35.0–55.0 g/l); alkaline phosphatase level, 120 U/l (normal range, 30–115 U/l) and serum β2-microglobulin level, 3,092 *μ*g/l (normal range, 0 to 2,300 *μ*g/l). The serum levels of the immunoglobulins IgG, IgA and IgM were all decreased and a serum protein electrophoresis test did not reveal any monoclonal peak. The 24-hour urinary protein excretion was 5.25 g and a monoclonal peak was detected by urine protein electrophoresis. Serum and urine immunofixation tests revealed positive results for λ-light chains. The white blood cell (WBC) count of the patient was 12.3×10E9/l (normal range, 4.0–10.0×10E9/l) and a bone marrow examination revealed 67.5% atypical plasma cells. Further radiographic studies included a normal brain computed tomography (CT) examination and a chest CT scan that exhibited multiple rib and vertebral bone destruction. A positron emission tomography (PET)-CT inspection indicated an uneven bone mass density, and four ribs on the left side were observed to be destroyed, with spindle-shaped soft tissue density shades and increased fluorodeoxyglucose (FDG) metabolism. On the right side, the eighth anterior rib was fractured. Following the investigations, the patient was diagnosed with λ-light chain MM [Durie-Salmon (DS) stage III, group A; International Staging System (ISS) stage I).

The patient received chemotherapy, which comprised a bortezomib-dexamethasone-cyclophosphamide regimen (1.3 mg/m^2^ intravenous bortezomib bolus on days 1, 4, 8 and 11; 20 mg/m^2^ intravenous dexamethasone on each day of the bortezomib administration, as well as the following day; and 300 mg cyclophosphamide on days 1–4), every 21 days, for three cycles. Four months subsequent to the end of the chemotherapy, the patient underwent autologous stem cell transplantation. Following the transplantation, the patient achieved a complete remission, with negative serum and urine immunofixation results. The patient was prescribed 100 mg thalidomide once a day for maintenance therapy, while the serum and urine immunofixation results of the patient were reviewed every six months, with further negative results.

The study was approved by the Ethics Committee of The First Affiliated Hospital of the Zhejiang University School of Medicine, and informed consent was obtained from the participant.

### Secondary treatment of the patient

**Figure 1.**
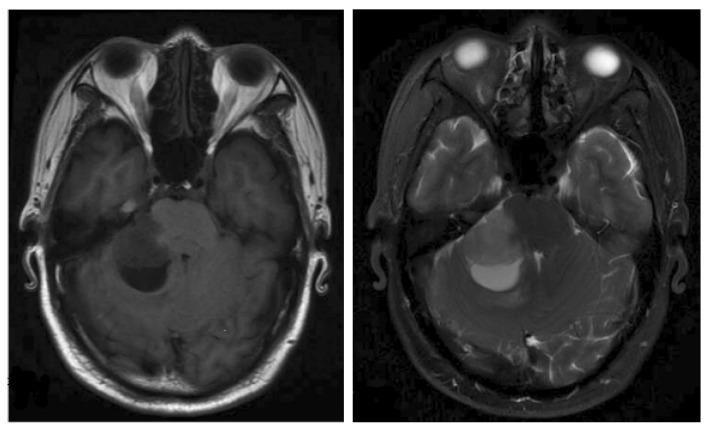
A large abnormal signal lesion was visible in the right cerebellar hemisphere with a size of ∼2.7×3.4 cm and an obscure boundary. T1-weighted imaging (T1WI; left panel) demonstrated low signals while T2-weighted imaging (T2WI; right panel) demonstrated a marginally higher signal intensity. Enhancement scanning revealed an inhomogeneous enhancement. A long T1 and long T2 signal revealed a cystic lesion and a patchy edema zone was visible in the peripheral lesion. The pontine was shifted marginally to the left, while the fourth ventricle was deformed and compressed.

Twenty-two months following the autologous stem cell transplantation, the patient presented with lower extremity weakness, an unsteady gait and right-sided facial numbness. The patient’s tongue deviated to the left when protruded and the finger-nose test result was positive. Immunoglobulins (including IgA, IgG and IgM) were all within the normal ranges and the serum and urine immunofixation tests were negative. There were no abnormal plasma cells in the bone marrow smears. Brain magnetic resonance imaging (MRI) revealed a mass (27.3×34.0×30.0 mm) in the right cerebellar hemisphere, with diffuse borders (Fig. 1). A PET-CT examination revealed tissue occupying the right cerebellopontine angle with abnormal increases in glucose metabolism, considered to be a malignant tumor, and multiple sites of bone destruction with some mild increases in glucose metabolism.

**Figure 2.**
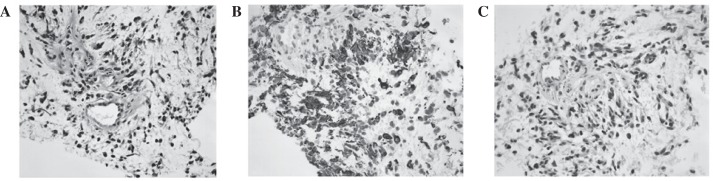
Hematoxylin and eosin staining of a pathological tissue paraffin section (magnification, ×40). (A) The tumor cells are astrocytes with round or elliptical nuclei and visible nuclear divisions. (B) Glial fibrillary acidic protein (GFAP) staining demonstrated positive expression, confirming an astrocyte tumor. (C) The tumor cells were negative for CD138 staining, confirming that the tumor was not a myeloma.

Initially, it was considered that the most likely diagnosis for the brain lesions was a relapse of the MM. In order to further investigate the tumor, a biopsy of the brain lesion was performed and the immunohistochemistry results of the intracranial needle biopsy-derived tumor cells were as follows: glial fibrillary acidic protein (GFAP), (+++); S-100, (+++); oligodendrocyte transcription factor-2 (Olig-2), (++); Wilms tumor-1 (WT-1), (+); isocitrate dehydrogenase-1 (IDH1), (+−); P53, (0++); O(6)-methylguanine-DNA methyltransferase (MGMT), (++); neurofilament (NF), (−); synuclein (Syn), (−); CD34, (−); CD68, (−); neuronal nuclei (Neu-N), (−); CD38, (−); CD138, (−); multiple myeloma oncogene 1 (Mum-1), (−); Kappa, (+−); Lambda, (+−); leukocyte common antigen (LCA), (−) and a Ki-67 labeling index of ∼10%, leading to the final diagnosis of an anaplastic astrocytoma (WHO grade III; Fig. 2).

Due to the large size and undesirable location of the lesion, the patient then received only localized irradiation, as well as temozolomide chemotherapy, and was discharged from hospital. One month subsequently, the patient became unconscious and succumbed to cerebral hernia caused by the rapid progression of the disease.

## Discussion

The coexistence of astrocytoma and MM is extremely rare. To the best of our knowledge, only four cases have been reported to date (4–7). In two of the four cases, the astrocytoma and MM were simultaneously diagnosed, while in the remaining two cases the astrocytomas developed subsequently to MM. By contrast, intracranial plasmacytomas affect a certain proportion of patients with MM, with 109 cases documented up to 2007 (8). In such cases, the intracranial plasmacytomas developed as primary manifestations of MM, following systemic MM, subsequent to complete remission, or during the disease progression. Due to the rarity of brain tumor developments in patients with MM, the complication was initially only recognized by neurological abnormalities; however, a further diagnosis was then made, using MRI as the main method to detect an intracranial tumor. MRIs of intracranial plasmacytomas generally appear as iso- to hyper-intense T1-weighted and iso- to hypo-intense T2-weighted images, with mild to marked contrast enhancement. Since intracranial plasmacytomas generally arise from cranial bone lesions or primary multiple dural myelomas, they usually appear in osseous or dural contact in MRIs, although isolated parenchymal involvements have been sporadically observed (9). By contrast, astrocytomas often infiltrate the white matter and may involve both hemispheres by spreading across the corpus callosum. Furthermore, they are usually hypo-intense in T1-weighted images and hyper-intense in T2-weighted images, exhibiting various degrees of edema and contrast enhancement (10). In the present case, the lesion was mildly hypo-intense in the T1-weighted and hyper-intense in the T2-weighted images, without osseous or dural contact, making a diagnosis of astrocytoma more likely than that of a CNS myeloma.

Imaging examinations are initially adequate for diagnosis; however, an unambiguous diagnosis depends on pathological examinations. For the specific diagnosis of intracranial plasmacytomas, a cerebrospinal fluid (CSF) examination remains the test most commonly used, as monoclonal plasma cells are easily detected, and pleocytosis, as well as increased protein levels, are very common in CNS myeloma CSFs (11). By contrast, the CSF examinations of patients with astrocytoma rarely reveal any abnormalities, unless tumoral hemorrhages or meningeal involvements have occurred. Despite this, when the results of the CSF examination are negative, a biopsy remains the only diagnostic method capable of establishing a definite diagnosis, which is important, due to the differences in the treatments for CNS myelomas and astrocytomas. With regard to CNS myeloma, radiotherapy (RT) has been demonstrated to be the most effective therapy, while novel agents, such as thalidomide, bortezomib and lenalidomide, have also resulted in good outcomes (12–14). By contrast, resection (or biopsy) and RT, along with temozolomide medication, remains the standard therapy with regard to anaplastic astrocytomas (15).

Since the incidence of astrocytomas in patients with MM is rare, a genetic aberration correlation between the two tumor types is difficult to establish. Bone marrow stromal cell antigen 2 (BST2) has been identified as a tumor marker for astrocytomas (16), and was first described as a marker for B-cell maturation. It is commonly expressed in five different human myeloma cell lines, as well as in monoclonal neoplastic plasma cells (17); however, the reason for this coexisting upregulation of a particular B-cell membrane protein in astrocytes is unclear. A further hypothetical correlation between the two tumor types is the commonly activated nuclear factor-κB (NF-κB) pathway and the excessive release of interleukin (IL)-6, which have been demonstrated to promote tumor cell proliferation and the invasion of astrocytoma and MM (18–20). In general, and based on current knowledge, it has been proposed that the development of secondary malignancies following MM is most likely a multifactorial process, involving the treatment, the MM and the patient, as well as environmental and behavioral factors (21).

In conclusion, this case report describes a patient, in whom an astrocytoma developed following MM, and emphasizes the importance of a differential diagnosis between astrocytoma and intracranial plasmacytoma. There is a requirement for clinicians to consider the possibility of a glioma, in addition to plasmacytoma, when a patient with MM presents with an intracranial space-occupying lesion.
